# Delivery of disulfiram into breast cancer cells using folate-receptor-targeted PLGA-PEG nanoparticles: in vitro and in vivo investigations

**DOI:** 10.1186/s12951-016-0183-z

**Published:** 2016-04-21

**Authors:** Hamidreza Fasehee, Rassoul Dinarvand, Ardeshir Ghavamzadeh, Mehdi Esfandyari-Manesh, Hanieh Moradian, Shahab Faghihi, Seyed Hamidollah Ghaffari

**Affiliations:** Tissue Engineering and Biomaterials Research Center, National Institute of Genetic Engineering and Biotechnology (NIGEB), Tehran, 14965/161 Iran; Nanotechnology Research Center, Tehran University of Medical Sciences, Tehran, Iran; Hematology, Oncology and Stem cell Transplantation Research Center, Shariati Hospital, Tehran University of Medical Science, Tehran, Iran; Department of Chemistry, Amirkabir University of Technology, Tehran, Iran; Faculty of Biomedical Engineering, Amirkabir University of Technology, Tehran, 15875/4413 Iran

**Keywords:** Disulfiram, PLGA nanoparticles, Folate receptor, Breast cancer cells, Apoptosis, Targeted drug delivery

## Abstract

**Background:**

A folate-receptor-targeted poly (lactide-co-Glycolide) (PLGA)-Polyethylene glycol (PEG) nanoparticle is developed for encapsulation and delivery of disulfiram into breast cancer cells. After a comprehensive characterization of nanoparticles, cell cytotoxicity, apoptosis induction, cellular uptake and intracellular level of reactive oxygen species are analyzed. In vivo acute and chronic toxicity of nanoparticles and their efficacy on inhibition of breast cancer tumor growth is studied.

**Results:**

The folate-receptor-targeted nanoparticles are internalized into the cells, induce reactive oxygen species formation, induce apoptosis and inhibit cell proliferation more efficiently compared to the untargeted nanoparticles. The acute and toxicity test show the maximum dose of disulfiram equivalent of nanoparticles for intra-venous injection is 6 mg/kg while show significant decrease in the breast cancer tumor growth rate.

**Conclusion:**

It is believed that the developed formulation could be used as a potential vehicle for successful delivery of disulfiram, an old and inexpensive drug, into breast cancer cells and other solid tumors.

**Graphical abstract:**
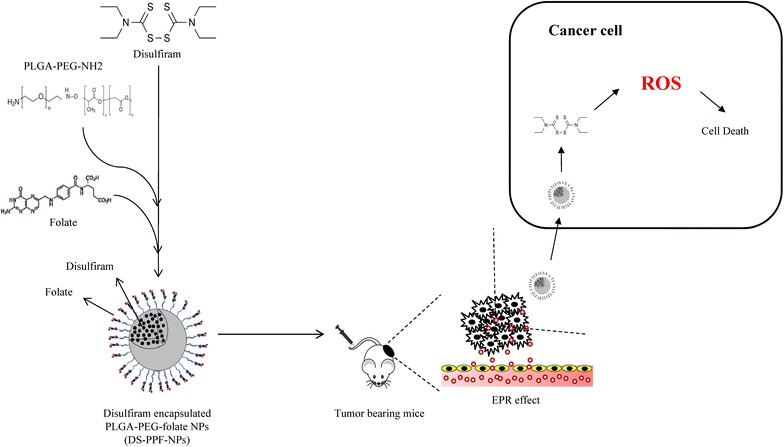
Disulfiram, an old and inexpensive drug, is encapsulated in folate-targeted PLGA-PEG nanoparticles and delivered into breast cancer cells using passive and active targeting to inhibit tumor growth in mice

## Background

Disulfiram (DS), a member of dithiocarbamate family with 297 Da molecular weight, is oral aldehyde dehydrogenase (ALDH) inhibitor that has been used in the treatment of alcoholism since 1940s [1–3]. During the last few years, a growing body of evidence from both in vivo and in vitro studies indicated that disulfiram has anticancer properties [2]. Anticancer properties of disulfiram have been demonstrated in pre-clinical models of hematological malignancies and solid tumors [4, 5]. Several mechanisms have been proposed for disulfiram induced cytotoxic effects. It has been shown that DS directly interacts with matrix metalloproteinase MMP-2 and MMP-9 and inhibits their proteolytic activity through a zinc chelating mechanism [2]. In vitro, DS has shown the ability to induce cytotoxic effects in cancer cells and inhibits the proteasome and NF- κB activities; as well as TNF-α-induced nuclear factor-KB (NF-κB) translocation [2]. DS has shown to be able to reduce P-glycoprotein (P-gp)-mediated drug resistance to vinblastine and colchicine by targeting P-gp itself [2]. DS is a highly reactive compound and its first metabolite, DDTC, react with many molecules and proteins in the cells. The cytotoxic effect of DS on the cancer cells has been also attributed to many other parameters including inhibition of DNA methyltransferase [6], reduction DNA replication [7], induction of oxidative stress [8], induction of mitochondrial membrane permeabilization, cell cycle arrest [8], inhibition of proteasome activity [1, 3, 7, 9, 10], inhibition of superoxide dismutase and increase intracellular reactive oxygen species (ROS) [1, 4]. The anticancer activity of DS is copper (Cu) dependent [11]. It means that the redox conversion of disulfiram is specific to Cu (II) and no other biological metal ions such as Fe (II or III) and Mn(III) [19]. Based on the chemical structure of DS it is speculated that the interaction between the drug and Cu (II) would be through its thiol groups [4]. In the gastrointestinal system DS is rapidly converted to bis (diethyldithiocarbamate) Cu complex. It could also be degraded into diethyldithiocarbamate (DDTC) during the absorption into the blood stream [4, 12].

Although the metabolisms and clinical pharmacology of DS are fairly understood; its potential application in cancer treatment is still hampered by its currently available oral formulation [12]. DS is extremely unstable in acidic gastric environment and is also rapidly degraded in blood stream [4, 12]. For example after an oral administration of a 500 mg of DS, its blood concentration would be still below the limit of detection [12]. Therefore, an efficient drug delivery system is essential for clinical application of DS in cancer treatment [12]. One strategy would be the encapsulation of DS into nanoparticles to protect it from degradation in blood system [12]. The targeted delivery of DS encapsulated nanoparticles into tumor cells could increase the drug accumulation at the tumor site [13–16]; and would enhance the endurance of the drug in blood circulation [13–16].

Poly (lactic-co-glycolic acid) (PLGA) has received considerable attention due to its attractive properties including biodegradability, biocompatibility, FDA approval for delivery systems, protection of drug from degradation, and possibility of sustained release [17]. PLGA-based nanoparticles have gained great interest in diagnostics and applications such as sustained drug release systems [18]. PLGA NPs have been used to develop proteins, peptides and nucleic acid based pharmaceutics. These NPs extravasate through the tumour vasculature and deliver the therapeutic agent into the cells by enhancing permeability and retention (EPR) effect [19]. In this study, a novel system for delivery of DS is developed using PLGA nanoparticles (NPs). Nanoprecipitation method is used for preparation of nanoparticles [18, 20]. In order to prevent the elimination of DS by liver and spleen from the blood stream, the surface of NPs is modified by hydrophilic polyethylene glycol (PEG) [4]. As we intend to deliver the nanoparticles into breast cancer cells mainly via receptor mediated endocytosis, the surface of NPs is also modified with folate. The folate receptor is overexpressed on the vast majority of cancer tissues, while its expression is limited in healthy tissues and organs. Folate receptors are highly expressed in epithelial, ovarian, cervical, breast, lung, kidney, colorectal and brain tumors [18]. Folate mediated cancer cell targeting is one of the most important methods for active targeting of therapeutic agents into cancer cells [21]. This is the first attempt for DS encapsulation into PEG-PLGA-folate NPs and its potential application in cancer treatment. It is presumed that the injection of NPs into the blood stream, protects the drug from rapid degradation, assists its delivery into specific tumor site and releases it in a sustained manner.

## Methods

### Materials

Poly (lactide-co-glycolide) (PLGA) (RG 504 H, acid terminated, lactide:glycolide 50:50, Mw: 38,000), Poly(ethylene glycol) (PEG)-bis-amine (Mn: 10,000), Poly(vinyl alcohol) (Mw: 31,000–50,000), folate, fluorescein-5-isothiocyanate (FITC), N,N′-Dicyclohexylcarbodiimide (DCC), Dichloromethane (DCM) and sulfo-N-hydroxysuccinimide (NHS) were obtained from Sigma-Aldrich (St. Louise, MO, USA); disulfiram, methylene chloride, diethyl ether, methanol and dimethyl sulfoxide (DMSO) was obtained from Merck (Darmstadt, Germany); Fetal bovine serum (FBS), DMEM media, PBS buffer, Trypsin/EDTA and penicillin–streptomycin were purchased from GIBCO (Maryland, USA); MTT (3-(4,5-dimethylthiazol-2-yl)-2,5- diphenyltetrazolium bromide) assay kit was obtained from Roche (Mannhein, Germany).

### Preparation and characterization of PLGA-PEG-folate conjugate

The synthesis of PLGA-PEG-Folate conjugate was previously described [18, 20]. Briefly, 2 g PLGA was dissolved in 15 mL of methylene chloride and the terminal carboxyl group of polymer was activated by addition of 207 mg DCC and 115 mg NHS to the solution at room temperature under nitrogen atmosphere for 24 h. The resultant solution was filtered to remove by-products from activated PLGA that precipitated by dropping into ice-cold diethyl ether. The activated PLGA was dried under vacuum and dissolved in 16 mL methylene chloride. The resultant solution was slowly added into PEG-bis-amine solution (200 mg/4 mL methylene chloride) in a molar ratio of 1:10 for activated PLGA/PEG-bis-amine. The reaction was carried out for 24 h under nitrogen atmosphere and the resultant solution was precipitated by the addition of ice-cold diethyl ether. The amine-terminated di-block copolymer PLGA-PEG-NH2, was obtained, filtered and vacuum dried.

**Fig. 1:**
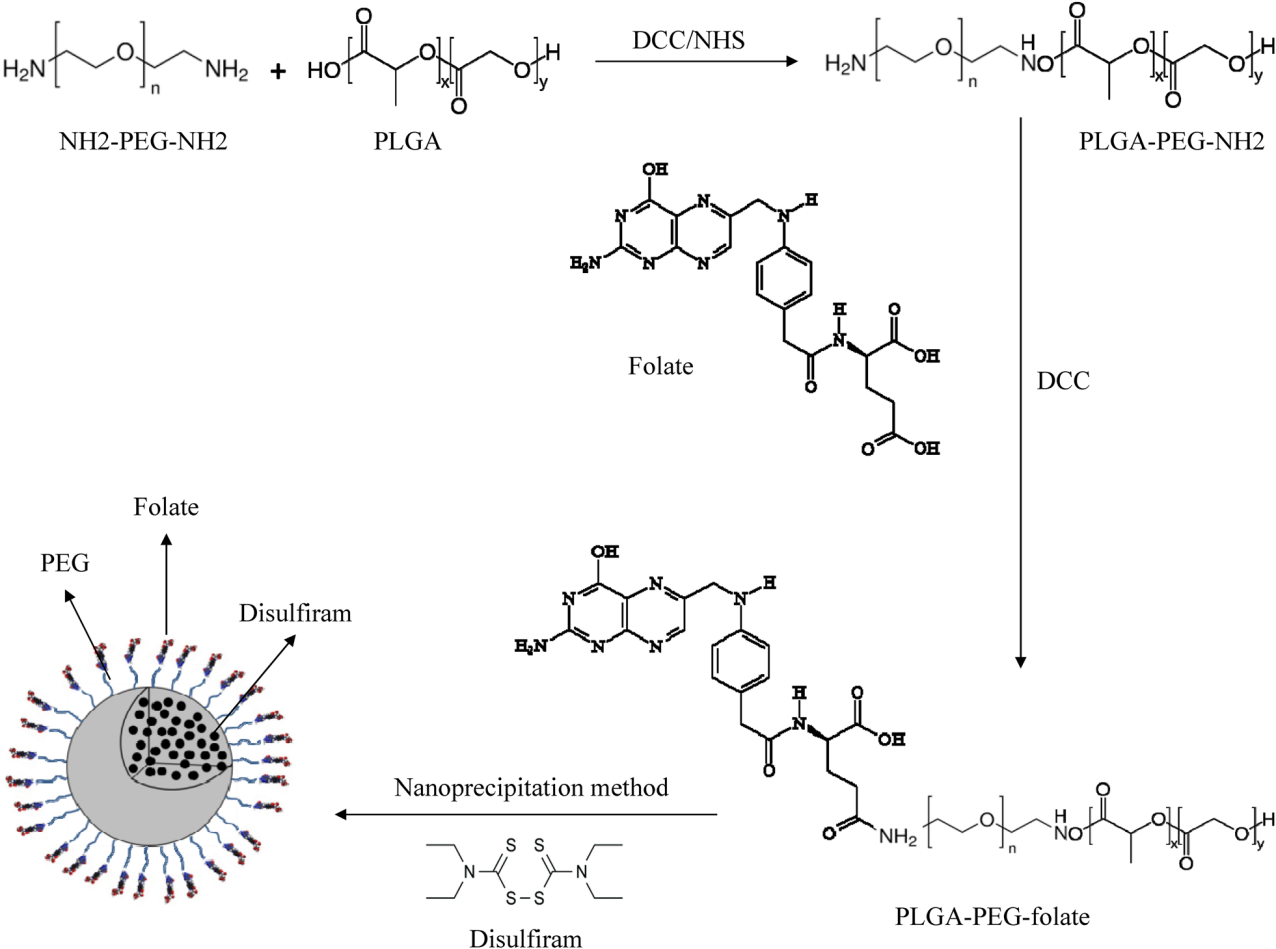
A representation of PLGA-PEG-folate synthesis and NPs preparation procedure

For conjugation of folic acid to PLGA-PEG-NH2 di-block copolymer, 500 mg of PLGA-PEG-NH_2_ (dissolved in 5 mL DMSO) was mixed with 13 mg folic acid and 13 mg DCC. The reaction was performed at room temperature for 7 h. The resultant solution was added to 100 mL cold methanol and filtered by a paper filter. The precipitated product was dried under vacuum and dissolved in 50 mL DCM for separation of free folate from conjugated folate. The solution centrifuged at 21,000*g* to isolate the free folate precipitation in DCM. The supernatant was dried under the vacuum (Fig. 1). The synthetic PLGA-PEG-folate was characterized using ^1^H NMR, FTIR and LC–MS analyses methods.

### Nanoparticle preparation

For preparation of nanoparticles, nanoprecipitation method was used [18]. Briefly, the appropriate amount of polymer (PLGA or PLGA-PEG-Folate) and disulfiram was dissolved in a DMSO to form a diffusing phase. In synthesis of disulfiram encapsulated PLGA-PEG-folate nanoparticle, a combination of PLGA-PEG-folate and PLGA ranging from (1:1) to (1:10) was chosen. The ratio of drug (disulfiram) to polymer (PLGA or PLGE-PEG-Folate) was 1:10 (w/w). The mixture was then added into the dispersing phase (PVA 0.5 % in water) using a syringe that positioned directly in the medium under moderate magnetic stirring (300 rpm, 10 min). The ratio of diffusing phase to dispersing phase was 1:20 (v/v). The freshly formed nanoparticles were obtained by dialyzing against water for 24 h. The nanoparticles were centrifuged at 20,000*g* for 15 min to remove DMSO and free disulfiram followed by several washing steps with distilled water. The purity of NPs was analyzed using spectrophotometry. The absence of DMSO in nanoparticle solution (in PBS) was confirmed at 265 nm, the absence of un-capsulated disulfiram was confirmed at 433 nm. The nanoparticles were then freeze-dried and kept at 4 °C.

### Characterization of nanoparticles

The mean particle size of the PLGA NPs was determined by dynamic light scattering using photon correlation spectroscopy. The measurements were performed using a Zetasizer Nano ZS (Malvern Instruments Ltd, Malvern, UK) equipped with a helium–neon laser at 25 °C and a scattering angle of 173°. The morphological examination of NPs was performed using a field emission scanning electron microscope at an accelerating voltage of 5 kV. A drop of diluted nanoparticle solution was placed onto a copper sheet and dried. For scanning electron microscopy (SEM) analysis, the surfaces of NPs were sputtered with gold in a vacuum before examination under the microscope.

### Drug loading and release behavior of NPs

To determine the drug loading and encapsulation efficiency of disulfiram in NPs, 150 mg of dried NPs was dispersed in 15 mL phosphate-buffered saline (PBS) solution (pH 7.4) to obtain a final concentration of 10 mg/mL. 10 μL of NPs suspension was added to 90 μL of DMSO to dissolve the PLGA and release the encapsulated disulfiram. The sample was vortexed for 30 s and 900 μL methanol was added to precipitate the PLGA polymer. The solution was mixed again, centrifuged and the supernatant was removed and analyzed by UV–Visible spectroscopy (433 nm) to estimate the amount of encapsulated disulfiram in NPs. A standard curve was prepared by making serial dilutions of disulfiram: cupper (1:1 molar ratio) in DMSO with specific concentrations [22]. The encapsulation efficacy (EE) was measured as the mass ratio of disulfiram encapsulated in NPs to that of used in the NPs preparation. The drug loading was determined as the weight ratio of disulfiram in NPs to the weight of NPs.

For the release behavior, NPs were dispersed in PBS (0.1 M pH: 7.4) at 37 °C and sealed in dialysis bag (MWCO: 12 kDa) and immersed in PBS with continuous shaking at 100 rpm. After 0, 24, 48, 72, 96 and 120 h, all release media were taken out and replenished with an equal volume of fresh PBS. The amount of released disulfiram was measured using HPLC method [14].

### MTT assay

The cytotoxicity of disulfiram encapsulated PLGA-PEG-folate NPs (DS-PPF-NPs), disulfiram encapsulated PLGA NPs (DS-P-NPs) and blank PLGA-PEG-folate NPs (PPF-NPs) on breast cancer cells (MCF7 and 4T1) was determined via the reduction of 3-(4, 5-dimethylthiazol-2-yl)-2, 5-diphenyl tetrazolium bromide (MTT, Sigma) to Formazan. Briefly, MCF7 and 4T1 (mice breast cancer cell line) cells were seeded at 5000/well in flat-bottom 96-well culture plate and incubated with different concentrations (0, 100, 200, 300, 400, 500, 750, 1000, 1250, 1500 and 2000 nM disulfiram equivalent) of DS-PPF-NPs, DS-P-NPs, PPF-NPs and free disulfiram for 24 h. After removing the media, cells were further incubated with MTT solution (5 mg/ml in PBS) at 37 °C and the untreated cells were used as control. After 3 h of incubation, the supernatant was removed and the cells were further treated with 100 µL of DMSO to dissolve the dark blue crystals of formazan and the absorbance was measured with an ELISA reader at 570 nm.

### Uptake of NPs by breast cancer cells

To quantitatively assess DS-PPF-NPs uptake by MCF7 cells, the fluorescein isothiocyanate (FITC) was added to the diffusing phase in NPs preparation process to obtain FITC labeled DS-PPF-NPs and FITC labeled DS-P-NPs. The FITC labeled NPs were incubated with MCF7 cells in 6-well plates (in 2 ml serum free basic medium/well) for 4 h. After removing the media, the cells were washed with PBS, trypsinized, centrifuged, and suspended in PBS for flowcytometry analysis (excitation/emission 350/461 nm). The instrument threshold for the negative control sample (untreated MCF7) was setup at ~1 % level. The percentage of cells exhibiting FITC-fluorescence beyond this threshold value was calculated as a function of NPs uptake [23]. The uptake of DS-P-NPs and DS-PPF-NPs was further performed in the presence and absence of folate. Briefly, MCF-7 cells were pretreated with or without folate (500 nM) overnight and seeded onto a 24-well plate (10,000 cells per well) with DMEM. The FITC labeled NPs were incubated with MCF-7 cells for 4 h. After several washing steps, the uptake of NPs was analyzed using flowcytometry. Moreover, the presence of NPs in breast cancer cells (MCF7) was examined by fluorescent microscopy. The nucleus of cells was stained using DAPI. Briefly, the cells were trypsinized, washed with PBS and fixed using 3.7 % formaldehyde for 10 min. The cells were washed and treated with 0.2 % Triton X-100 for 5 min and the cells treated with an appropriate amount of DAPI labeling solution (1:5000 DAPI in PBS) for 5 min. After washing with PBS cells were analyzed with fluorescent microscopy.

### Quantification of apoptosis using PI/Hoechst 3342 staining

To explore the effect of NPs on breast cancer cells apoptosis and to determine the efficiency of targeting agent (Folate) for induction of apoptosis, Chromatin Condensation/Dead Cell Apoptosis assay was performed [24]. Briefly, MCF7 cells were treated with DS-PPF-NPs, DS-P-NPs, PLGA NPs (without disulfiram) and PPF-NPs (without disulfiram) for 24 h with drug concentration of 250 nM disulfiram equivalent. Apoptosis was quantified by Chromatin Condensation/Dead Cell Apoptosis Kit using Hoechst 33342 and Propidium iodide (PI) for flowcytometry. First, the treated cells were washed with cold phosphate-buffered saline (PBS) and the cell density was adjusted to 5 × 10^5^ cells/mL. Then 1 μL of the Hoechst 33342 stock solution (5.0 mg/ml solution in water) and 1 μL of the PI stock solution (1.0 mg/ml solution in water) were added to 1 mL of cell suspension. After 20 min incubation on ice, the stained cells were evaluated by flowcytometry (PartecPasIII, Germany) using excitation/emission 350/461 and 535/617 nm for Hoechst 33342 and PI, respectively. The data was collected and analyzed with FlowMax software.

### Clonogenic assay

To determine the effect of disulfiram encapsulated NPs and free disulfiram on colony forming capability of breast cancer cells, clonogenic assay was performed [16]. Briefly, MCF7 cells (5 × 10^4^/well) were cultured in 6-well plates overnight and exposed to 500 nM of free disulfiram, DS-PPF-NPs and DS-P-NPs with drug concentration of 250 nM disulfiram equivalent for 12 h. The cells were collected and cultured in 6-well plates containing drug-free medium at a density of 250/well. The clonogenic cells were considered as the ones with the ability to form a colony consist of at least 50 cells after 10 days of culture.

### Measurement of ROS activity

To determine the oxidative stress of the treated cells, dichloro-dihydro-fluorescein diacetate (DCFH-DA) assay was performed. This test is a quantitative method for detection of intracellular production of ROS. DCFH-DA (uncharged) is taken up by cells and cleaved by nonspecific esterases to create DCFH (charged) which is further oxidized by ROS to make DCF which is highly fluorescent. After incubation with desired concentration of disulfiram, DS-P-NPs and DS-PPF-NPs for 24 h, breast cancer cells (MCF7) were exposed to 10 μM DCFH-DA and incubated for 30 min at 37° C. The cells were washed twice with PBS and resuspended in PBS. Finally, fluorescence intensity of the samples was detected by fluorescence spectrophotometer (Cary Eclipse, USA) with excitation at 485 nm and emission at 530 nm [24].

### Acute and chronic toxicity tests

Thirty-six 5-week-old male BALB/c mice weighing an average of 25 g (Institute of Pasture, Tehran, Iran) were selected to study the acute and chronic toxicity of DS-PPF-NPs. In acute toxicity test, 100 mg/kg of DS-PPF-NPs was given as starting dose. The animals were divided into 5 groups, each consisting of 4 mice as follows:

Group 1: control (200 μL of PBS).

Group 2: 2000 mg/kg DS-PPF-NPs (equivalent to 100 mg/kg disulfiram).

Group 3: 225 mg/kg DS-PPF-NPs (equivalent to 12.5 mg/kg disulfiram).

Group 4: 120 mg/kg DS-PPF-NPs (equivalent to 6 mg/kg disulfiram).

Group 5: 60 mg/kg DS-PPF-NPs (equivalent to 3 mg/kg disulfiram).

In animal study, the NPs administered intravenously into the tail vein of animals except the 2000 and 225 mg/kg NPs which administered using intraperitoneal routeas these doseswere too high to be administer intravenously. After 24 h, animals were sacrificed and the abnormal hematological indices were screened.

According to the results of acute toxicity test, the chronic toxicity test was performed using the following groups:

Group 1: control (120 mg/kg PPF-NPs) (NPs without disulfiram).

Group 2: 120 mg/kg DS-PPF-NPs (equivalent to 6 mg/kg disulfiram).

Group 3: 60 mg/kg DS-PPF-NPs (equivalent to 3 mg/kg disulfiram).

Group 4: 30 mg/kg DS-PPF-NPs (equivalent to 1.5 mg/kg disulfiram).

Group 5: 15 mg/kg DS-PPF-NPs (equivalent to 0.75 mg/kg disulfiram).

For chronic toxicity test, the drug was administered for 7 consecutive days. In all groups, the drug was dissolved in PBS (200 μL) and administered intravenously via 1 ml syringe. Animals were sacrificed 7 days after drug administration and the abnormal hematological indices were screened. The anesthesia of animals was performed using ketamine/xylazin (8/1 mg per 100 g body weight of animals) and the blood samples were directly taken from animals heart using 1–3 mL syringe. The animals were then sacrificed using diethyl ether anesthesia.

Blood samples were taken for clinical chemistry tests. Total leukocyte count (WBC), erythrocyte count (RBC), platelets (Plt), hemoglobin (Hgb), hematocrit (Hct), mean cell volume (MCV) and mean corpuscular hemoglobin concentration (MCHC), were measured using an animal blood counter (Celltac; Nihon Kohden, Tokyo, Japan). Plasma urea nitrogen (Urea), creatinine (Cr), and glucose (Glu) were determined using CCX System (CCX WB; Nova Biomedical, USA). Plasma alkaline phosphatase (ALP), albumin (ALB), direct bilirubin (DBil) were also measured (Autoanalyser Model Biotecnica, BT3500, Rome, Italy).

### Inhibition of breast cancer growth in mouse model

The effect of disulfiram, DS-P-NPs and DS-PPF-NPs on suppression of breast cancer tumor growth in vivo was performed. Five-week-old female BALB/c mice (Institute of Pasture, Tehran, Iran) were housed according to the relevant laws and guidelines for animal care set forth by institutional laboratory animal care. The 4T1 tumor model was generated by an orthotropic injection consists of 1 × 10^6^ cells in 50 μL PBS into the mammary fat pad of the mice. After the tumor volume was reached to 200–250 mm^3^, tumor bearing mice were randomly divided into four groups (n = 3).

Group 1: control (200 mg/kg of blank NPs in 200 μL of PBS).

Group 2: control (10 mg/kg of free disulfiram in PBS).

Group 3: 200 mg/kg DS-P-NPs in PBS (equivalent to 10 mg/kg disulfiram).

Group 4: 200 mg/kg DS-PPF-NPs in PBS (equivalent to 10 mg/kg disulfiram).

The samples were injected into the tail vein of each mouse for 2 weeks (every 3 days). At various time intervals tumor volume was measured by a vernier caliper and calculated using the following equation:

V = (L × W2) × 0.5 where L is the length and W is the width of the tumor [25, 26].

Animals were sacrificed 2 weeks after drug administration using diethyl ether anesthesia and the size and weight of tumors were analyzed.

All the ethical and the humanity considerations were performed according to the Helsinki humanity research declaration during the experiments and the euthanasia of the animals. All the animals’ experiments were approved by the Ethics Committee of the Tehran University of Medical Sciences. All experiments were performed on BALB/c mice housed in cages in a temperature controlled room (23 ± 2 °C) with a light/dark cycle of 12/12 h.

### H&E staining

For H&E staining, paraffin-embedded sample slides were deparaffinized, hydrated, and then stained with hematoxylin for 1 min. After rinsing, the slides were stained with eosin for 1 min, rinsed, and sealed with coverslips and analyzed using optical microscopy [25, 26].

### Statistical analysis

All the results are the mean ± SD of three independent experiments. The significance of differences (*p* < 0.05) between experimental variables was determined by the use of two-tailed Student’s test (SPSS.16). The statistical significance was indicated by *p* < 0.05.

## Results

### Characterization of PLGA-PEG-folate conjugate

**Fig. 2:**
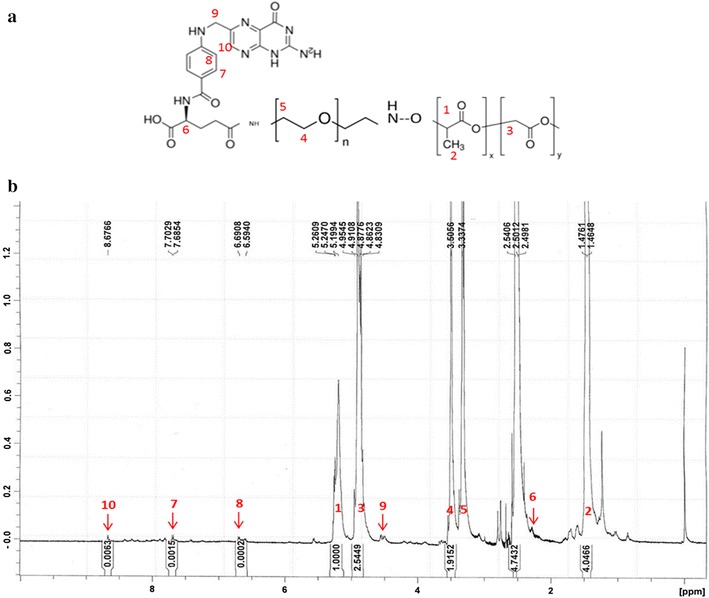
The structure of PLGA-PEG-folate and its proton’s position (**a**), the ^1^H NMR spectra of PLGA-PEG-folate (**b**). The peak positions in spectra correspond to the proton positions in PLGA-PEG-folate structure

^1^H NMR spectra of PLGA-PEG-folate was obtained using the Avence^®^ (500 MHz; CDCl3) and is presented in Fig. 2. The folate group of polymer shows typical peaks of 6, 7, 8, 9, 10 (2.34, 7.68, 6.60, 4.51 and 8.67 ppm) which represents the aromatic and pteridine protons of the folate. The typical peaks of PLGA at 5.20–5.40, 4.60–4.90 and 1.50 ppm as well as PEG at 3.33 and 3.50 ppm were also detected [18].

**Fig. 3:**
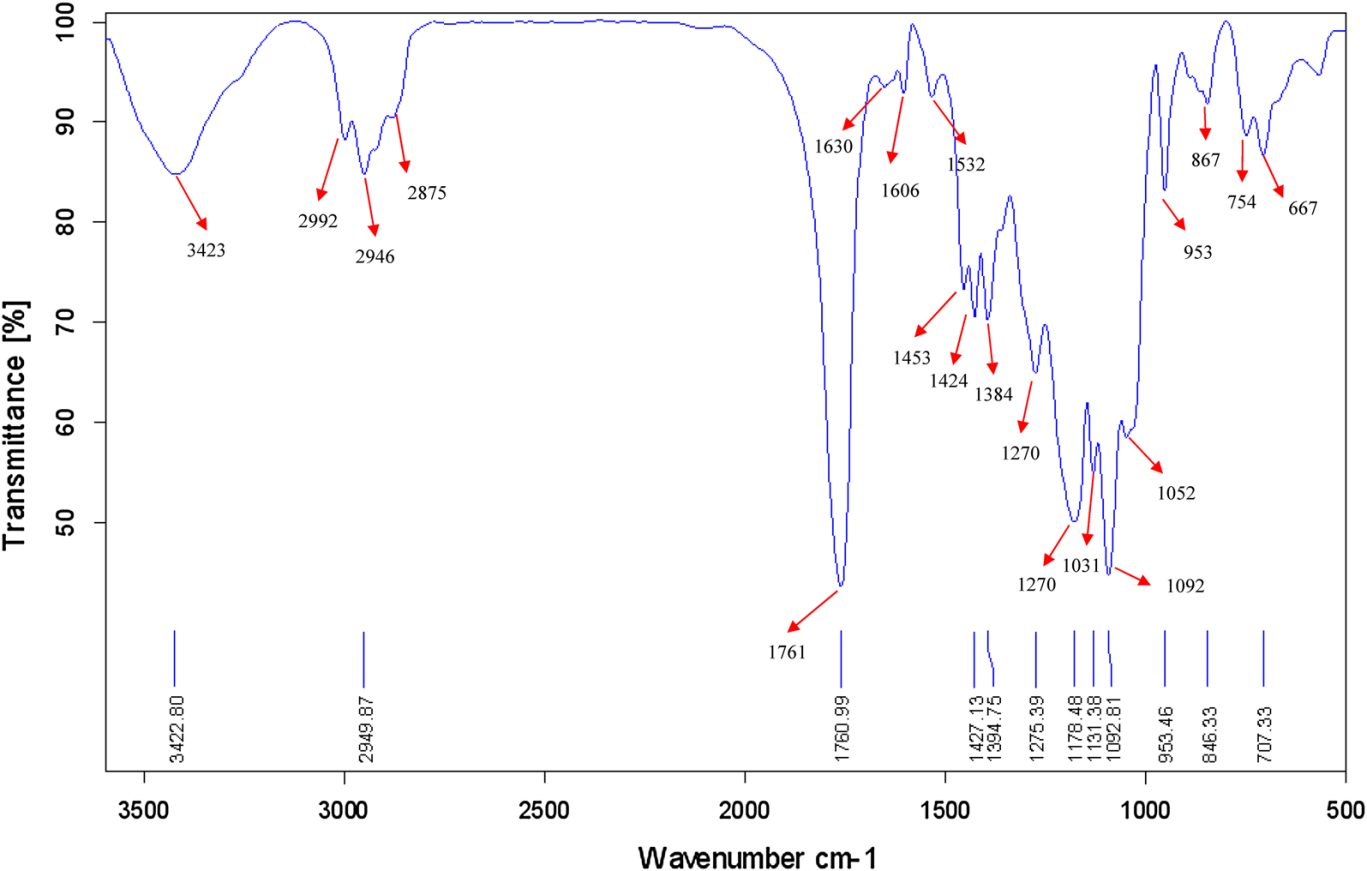
The FTIR spectra of PLGA-PEG-folate

FTIR studies were carried out to confirm the presence of amide linkage in polymer. Characteristic FTIR absorption bands of folate in PLGA-PEG-folate were observed at 1606 (Aromatic C = C bending) and 3423 (Amine N–H stretch). The presence of amide bond C = O stretch) between PLGA-PEG and PEG-folate linkages were observed at 1630 cm^−1^. The presence of PEG can be seen in 1091 cm^−1^ (C–O–C) and 1630 cm^−1^ (C = O). The presence of PLGA was confirmed by 2946 and 2992 cm^−1^ peaks (Alkyl C-H stretch) (Fig. 3).

**Fig. 4:**
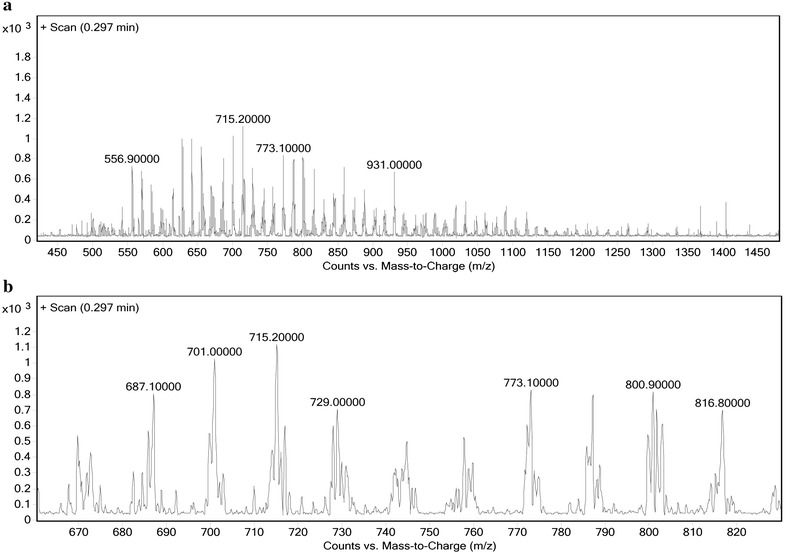
The LC–MS spectrum of PLGA. The Counts vs. Mass-to-Charge (m/z) from 450 to 1450 (**a**) and 670 to 820 (**b**) is presented

**Fig. 5:**
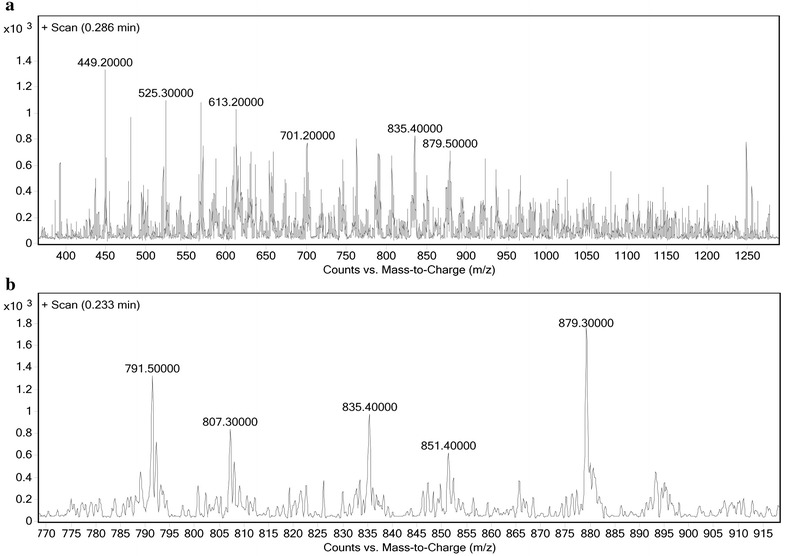
The LC–MS spectrum of PLGA-PEG-folate. The Counts vs. Mass-to-Charge (m/z) from 400 to 1250 (**a**) and 770 to 915 (**b**) is presented

LC–MS analysis was carried out to compare the length of PLGA and synthetic polymer (PLGA-PEG-folate). The LC–MS spectrum of PLGA and PLGA-PEG-folate is presented in Figs. 4 and 5, respectively.

Two adjacent peaks are chosen and the number of polymer cleavage is calculated as follow:

$$\rm{n} = \frac{\rm{b} - 1}{\rm{a} - \rm{b}}$$

where (n) indicates the number of polymer cleavages, (a) indicates the peak with greater scale and (b) indicates the peak with lower scale. The molecular weight of polymer is calculated as follow:

$$\rm{M} = \rm{an} - \rm{n}$$

Where (M) indicates the molecular weight of polymer chain. The molecular weight of PLGA and PLGA-PEG-folate were calculated 35.2 and 44.3 KDa, respectively. The molecular weight increase of PLGA-PEG-folate compared to PLGA could be due to the addition of PEG chain and folate group. These information confirms that the synthesis of PLGA-PEG-folate was successfully accomplished. In order to purify the synthesized polymer and reduce the contaminants, several steps of washing and dialysis were performed. Finally, the absence of impurities such as solvents and byproducts was confirmed by spectrophotometry.

### Characterization of nanoparticles

Two types of NPs were synthesized; disulfiram encapsulated PLGA NPs (DS-P-NPs) and disulfiram encapsulated PLGA-PEG-folate NPs (DS-PPF-NPs). A combination of PLGA-PEG-folate and PLGA ranging from (1:1) to (1:10) was chosen for synthesis of DS-PPF-NPs. The size of NPs was subsequently analyzed to have a final diameter size of ~200 nm. The results show that with a ratio of (1:5) the size of NPs was 204 nm so this ratio was chosen for synthesis of DS-PPF-NPs.The physicochemical characteristics of NPs are represented in Table 1. The carboxylic groups of PLGA create negative surface charge on the surface of NPs as detected with the zeta potential that showed a more negative value for DS-P-NPs rather than DS-PPF-NPs. This is logical since a ratio of (1:5) of PLGA-PEG-folate was used for preparation of DS-PPF-NPs. The addition of folate on the surface of NPs would reduce the overall negative charge of NPs as the carboxylic end of PLGA is partly covered with the addition of PEG-folate.

**Table 1:** Average particle size, drug loading, encapsulation efficiency and zeta potential of DS-P-NPs and DS-PPF-NPs

NPs	Drug loading (%)	Encapsulation efficiency (%)	Average particle size (nm)	Zeta potential
DS-P-NPs	5.35 ± 0.03	58.85 ± 1.01	165	−11.22 ± 0.84
DS-PPF-NPs	5.42 ± 0.06	59.62 ± 0.66	204	−5.24 ± 0.62

**Fig. 6:**
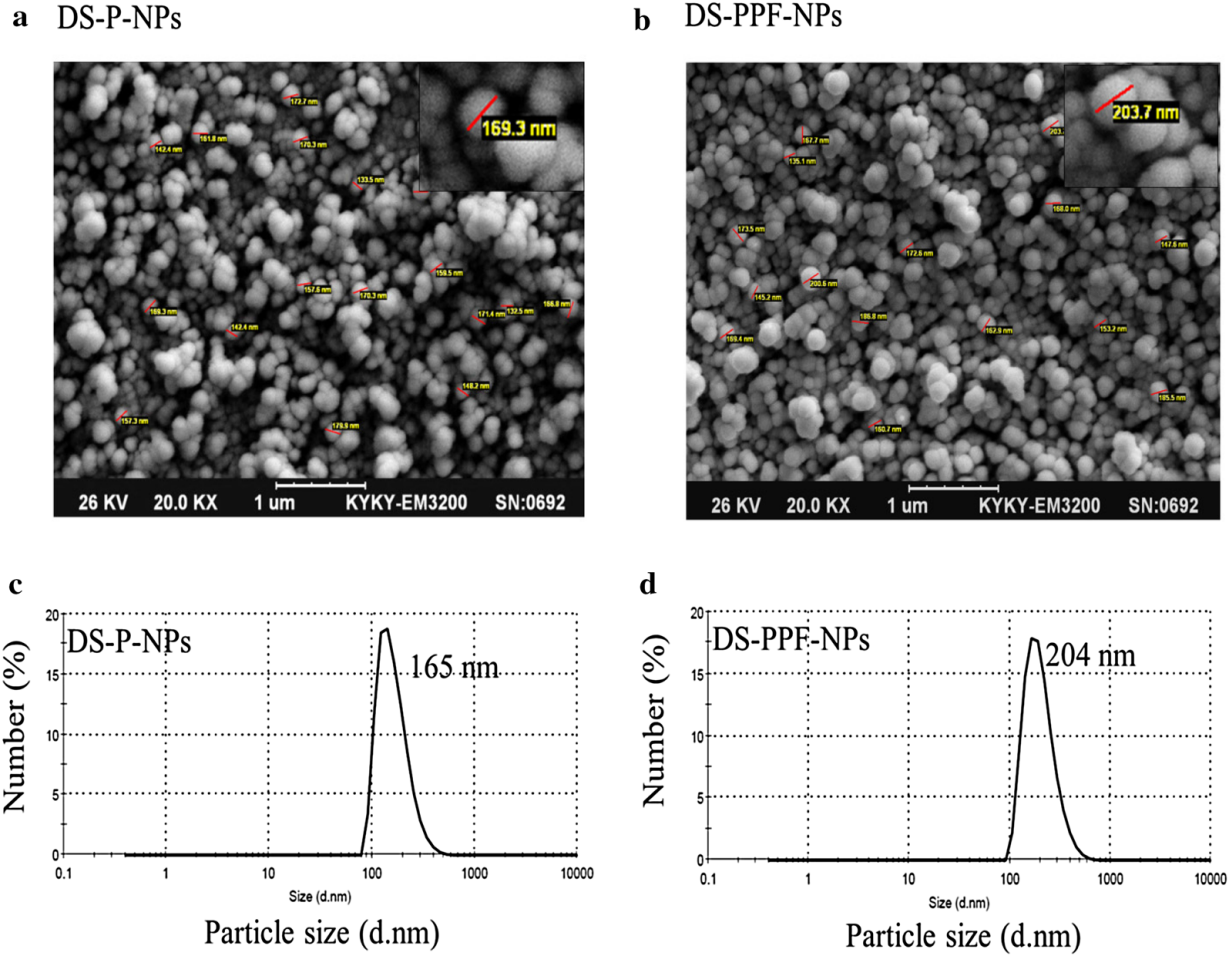
SEM image of DS-P-NPs (**a**), DS-PPF-NPs (**b**), average particle size of DS-P-NPs (**c**), average particle size of DS-PPF-NPs (**d**)

To determine the size range of NPs, SEM (Fig. 6a, b) and zeta sizer (Fig. 6c, d) were used. The results showed an average size of 165 nm (PDI: 0.32) for DS-P-NPs whereas the average particle size of DS-PPF-NPs was around 204 nm (PDI: 0.20).

### Drug loading, encapsulation efficiency and release profile

The results of drug loading and encapsulation efficiency of NPs are presented in Table 1. The drug loading into the NPs was 5.35 ± 0.03 and 5.42 ± 0.06 % for DS-P-NPs and DS-PPF-NPs, respectively. Encapsulation efficiency of NPs was 58.85 ± 1.01 and 59.62 ± 0.66 % for DS-P-NPs and DS-PPF-NPs, respectively. The dialysis against water was used as the final step in preparation of PLGA NPs. The dialysis was carried out for 24 h to ensure no trace of DMSO in NPs. It is assumed that the low encapsulation efficiency of disulfiram into the PLGA NPs is the result of disulfiram release from NPs in the dialysis period.

**Fig. 7:**
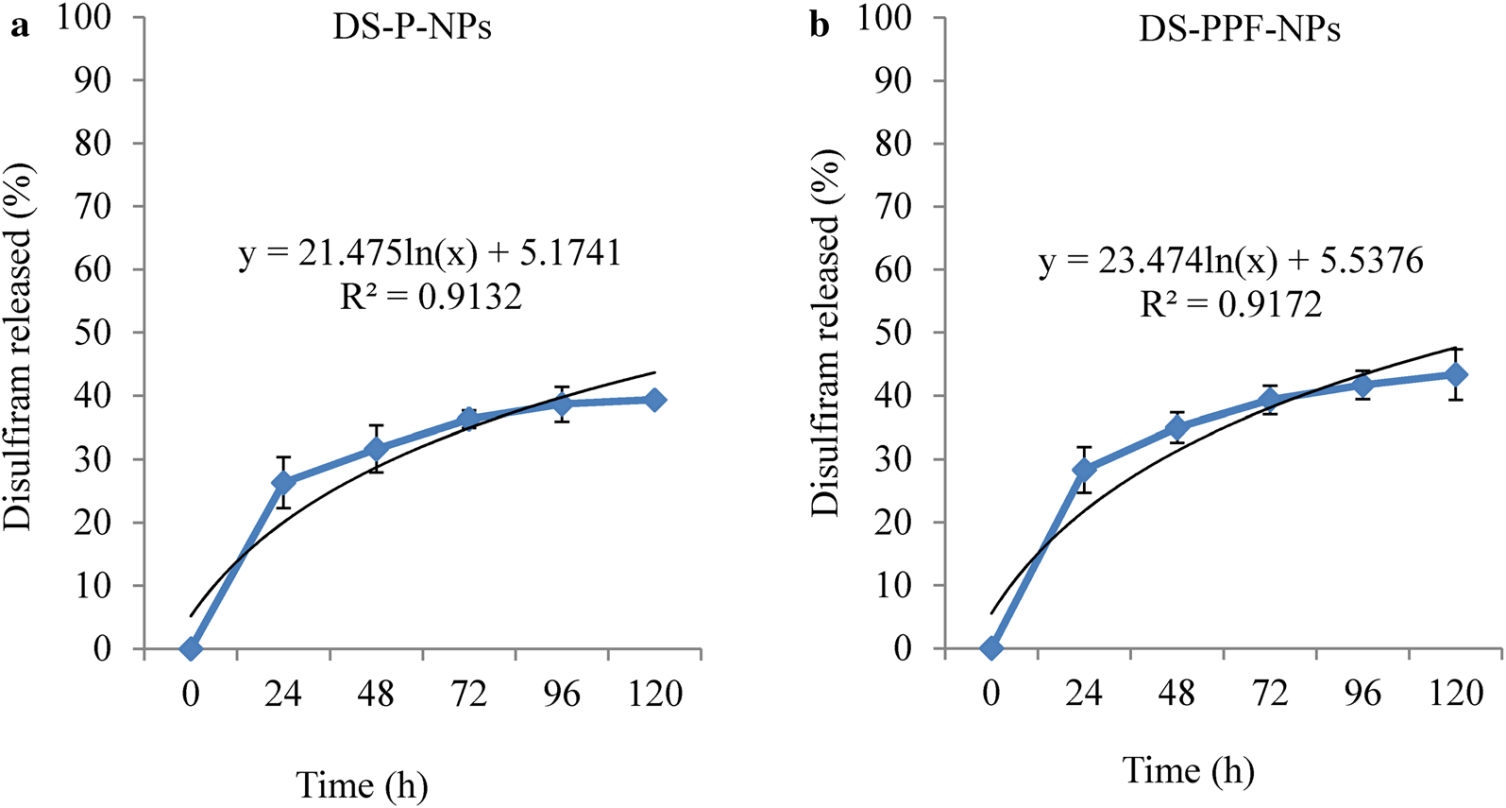
In vitro disulfiram’s release curves from DS-P-NPs (**a**) and DS-PPF-NPs **(b**)

Considering that the controlled and continuous drug release is essential for the success of pharmaceuticals; we further examined the drug release profile of the NPs. Both DS-P-NPs and DS-PPF-NPs showed an initial burst release effect. During the first sampling time (24 h), disulfiram release was 26.33 and 28.33 % for DS-P-NPs and DS-PPF-NPs, respectively. After 120 h, 39.3 and 43.3 % of encapsulated disulfiram released from DS-PPF-NPs, respectively (Fig. 7a, b).

### Uptake of DS-PPF-NPs by cells (MCF7)

**Fig. 8:**
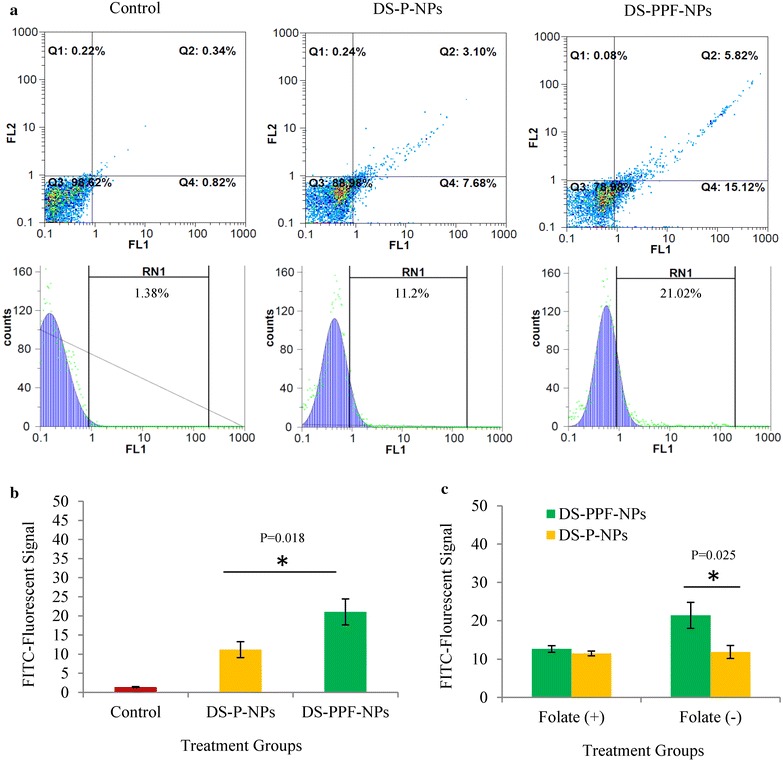
The results of flowcytometery analysis indicating the uptake of FITC labeled DS-P-NPs and FITC labeled DS-PPF-NPs into MCF7 cells (**a**); the comparison between uptake rate of FITC labeled DS-P-NPs and FITC labeled DS-PPF-NPs. (**b**); the comparison between uptake of DS-P-NPs and DS-PPF-NPs by MCF-7 cells in the absence (−) and presence (+) of folate (**c**); The values are mean ± SD. *Indicates the significant difference between DS-P-NPs and DS-PPF-NPs

To investigate whether disulfiram-encapsulated NPs were internalized into the breast cancer cell line MCF7, cellular uptake of FITC labeled DS-P-NPs and FITC labeled DS-PPF-NPs were analyzed by flowcytometry and fluorescent imaging microscopy. As shown in Fig. 8a, the cells incubated with FITC labeled DS-P-NPs and FITC labeled DS-PPF-NPs exhibited 11.2 and 21.02 % fluorescent intensity, respectively while untreated control cells didn’t show any FITC-fluorescent signal (RN1 = 1.38 %). The uptake rate of FITC labeled DS-PPF-NPs was significantly (p < 0.05) higher than FITC labeled DS-P-NPs (Fig. 8b).

Figure 8c shows that in the presence of folate, there is no difference between uptake of DS-P-NPs and DS-PPF-NPs by MCF-7 cells. However when folate eliminated from the medium, the uptake of DS-PPF-NPs was increased compared to DS-P-NPs. It is documented that PLGA NPs could cross the cell membrane even without targeting agents [27]. However, it is assumed that the addition of folate agent on the surface of NPs would increase the uptake of NPs by FR positive cells.

**Fig. 9:**
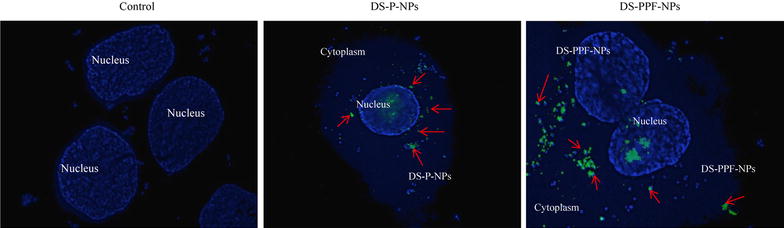
The fluorescent imaging of MCF7 cells containing FITC labeled DS-P-NPs and FITC labeled DS-PPF-NPs

The Uptake of FITC labeled DS-P-NPs and FITC labeled DS-PPF-NPs were also evaluated by fluorescent imaging microscopy (Fig. 9). The results showed that both FITC labeled DS-P-NPs and FITC labeled DS-PPF-NPs were internalized into the MCF7 cells effectively.

### Apoptosis induction by NPs

**Fig. 10:**
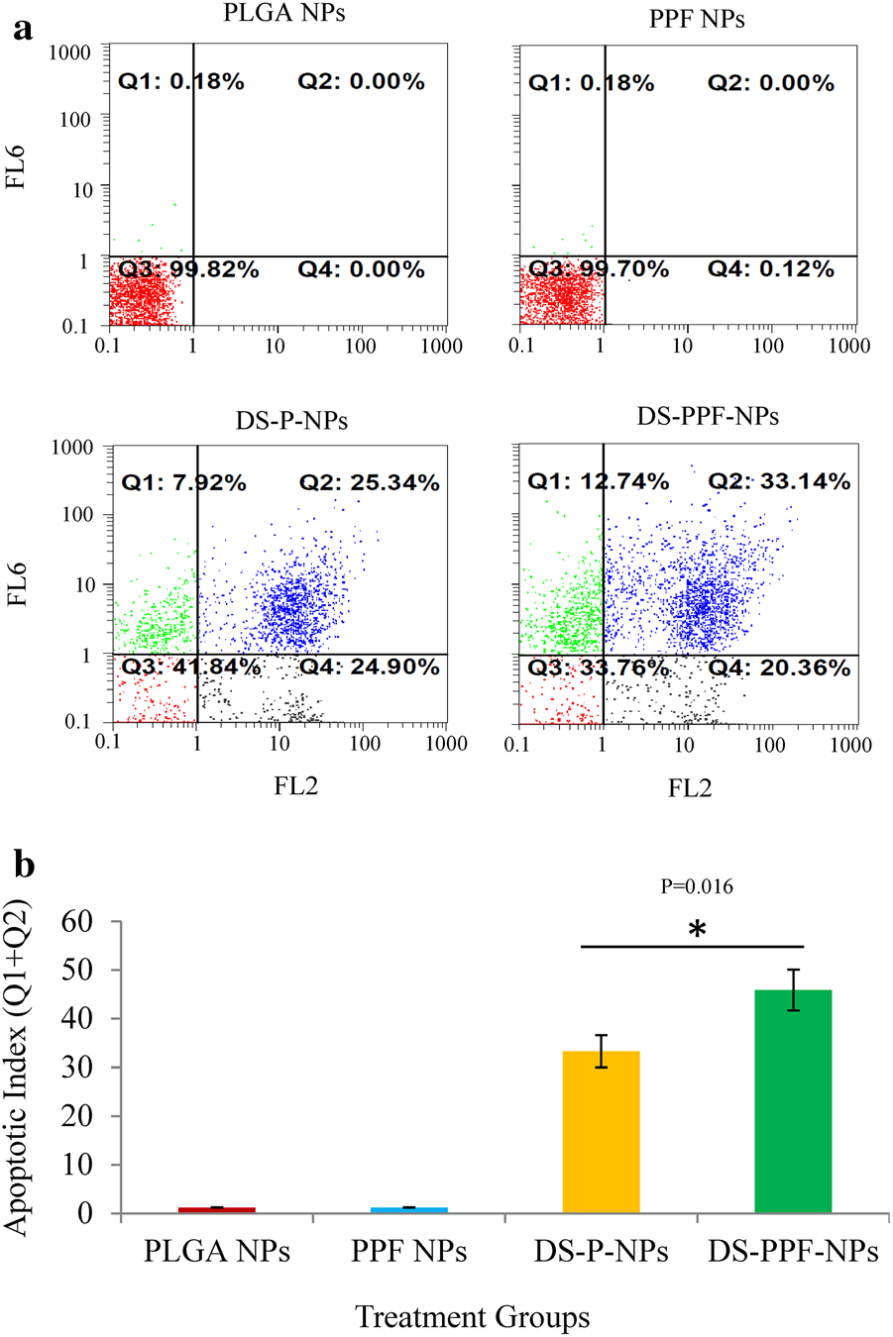
Apoptosis assay by Hoechst 33342 and PI using flowcytometery on MCF7 cells treated with PLGA NPs, PPF-NPs, DS-P-NPs and DS-PPF-NPs. FL2: PI, FL6: Hoechest 33342 (**a**); the comparison between apoptotic index of PLGA NPs, PPF-NPs, DS-P-NPs and DS-PPF-NPs (**b**); the values are mean ± SD. *Indicates the significant difference between DS-P-NPs and DS-PPF-NPs

Apoptosis assay was performed by Chromatin Condensation/Dead Cell Apoptosis Kit. The double stain Hoechst 33342 and PI render is a rapid and convenient assay for apoptosis and it is based on the fluorescent detection of the compacted state of chromatin in apoptotic cells [32]. Hoechst 33342 stains the condensed chromatin in apoptotic cells (Q4) more radiantly than the chromatin in normal cells (Q3) while PI is only permeable to dead cells (Q1 and Q2). The result showed that total early and late apoptosis were 33.2 % and 45.8 % for DS-P-NPs and DS-PPF-NPs, respectively (Fig. 10a). Neither PLGA NPs nor PPF-NPs cause apoptosis in MCF7 cells. The difference between DS-P-NPs and DS-PPF-NPs in apoptosis index was significant at p < 0.05 (Fig. 10b).

### ROS activity and colony formation

**Fig. 11:**
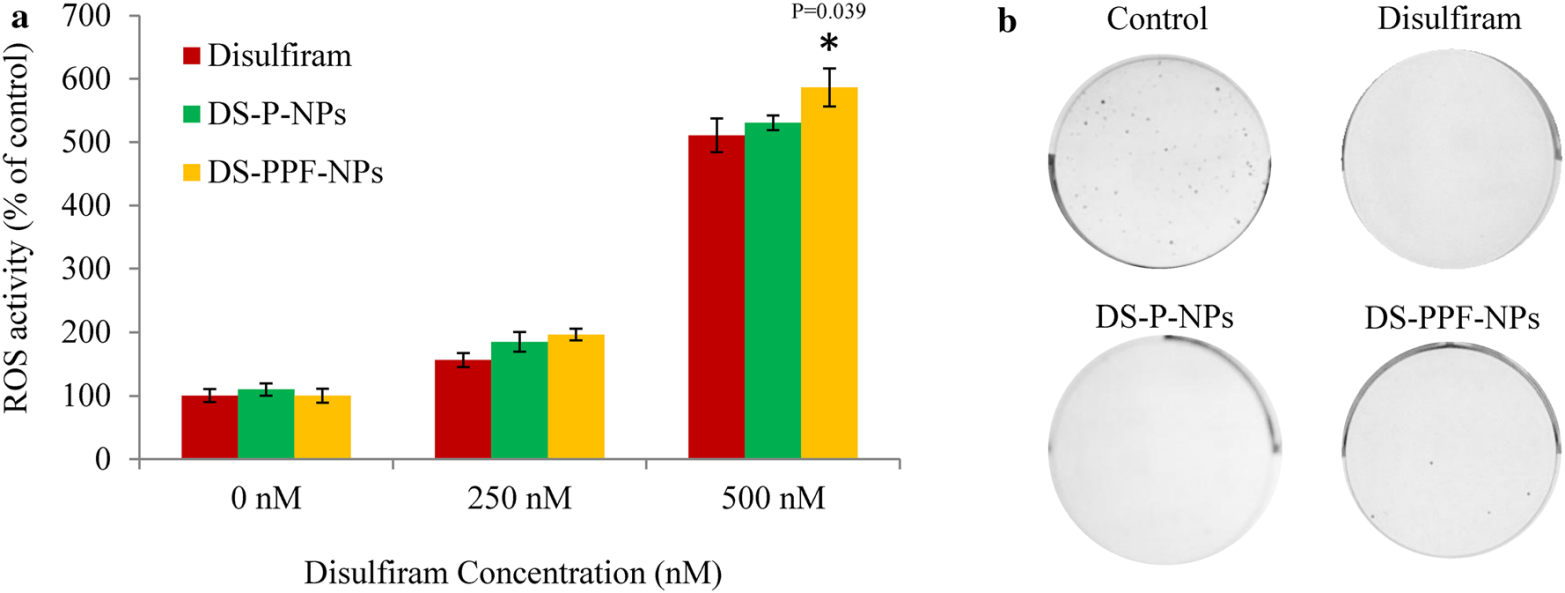
The ROS activity in MCF7 cells treated with free disulfiram, DS-P-NPs and DS-PPF-NPs (**a**); the effect of free disulfiram, DS-P-NPs and DS-PPF-NPs on the clonogenicity of MCF7 cells (**b**); the values are mean ± SD. *Indicates the significant difference between DS-PPF-NPs and free disulfiram

Induction of ROS generation has been postulated to be an essential mechanism by which disulfiram initiate the apoptotic process. The ROS activity of MCF7 cells incubated with free disulfiram, DS-P-NPs and DS-PPF-NPs were investigated. The result showed that with disulfiram concentration of 250 nM, the intracellular ROS level was increased to 156, 184 and 196 % when MCF7 cells were treated with free disulfiram, DS-P-NPs and DS-PPF-NPs, respectively (Fig. 11a). At the concentration of 500 nM, the intracellular ROS level of MCF7 cells treated with free disulfiram, DS-P-NPs and DS-PPF-NPs was increased to 510, 530 and 586 %, respectively. The results showed significant increase in ROS activity in DS-P-NPs and DS-PPF-NPs groups compare to free disulfiram at 250 nM (p < 0.05). At 500 nM concentration of disulfiram, the ROS activity in DS-PPF-NPs was significantly higher compare to DS-P-NPs and free disulfiram (p < 0.05).

To examine the ability of DS-P-NPs and DS-PPF-NPs to induce reproductive death in breast cancer cells, MCF7 cells were exposed to the free disulfiram, DS-P-NPs and DS-PPF-NPs at 250 nM concentration of disulfiram equivalent for 12 h; the treated cells were collected and cultured in drug-free medium for 14 days. The MCF7 colony number for free disulfiram and both disulfiram encapsulated NPs (DS-P-NPs and DS-PPF-NPs) was totally eradicated (Fig. 11b).

### MTT assay

**Fig. 12:**
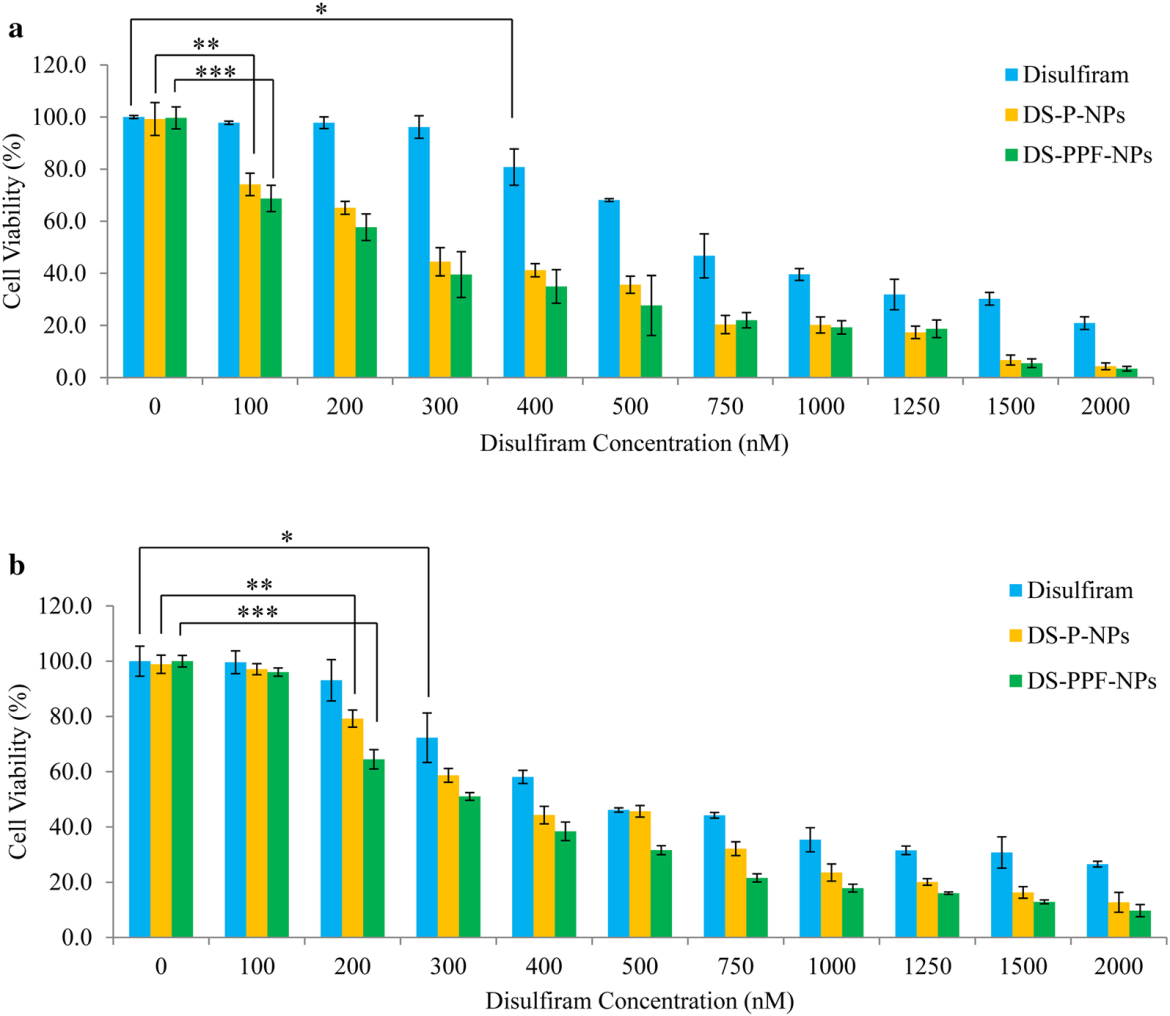
Cytotoxicity of free disulfriam, DS-P-NPs and DS-PPF-NPs on MCF7 (**a**) and 4T1 cells (**b**); the values are mean ± SD. *p < 0.05 (for disulfiram); **p < 0.05 (for DS-P-NPs); ***p < 0.05 (for DS-PPF-NPs)

For analyzing the in vitro cytotoxic effect of DS-P-NPs and DS-PPF-NPs, MTT cell proliferation assay was performed using two breast cancer cell lines including MCF7 and 4T1. In order to analyze the potential cytotoxicity of NPs without drug, PPF-NPs (blank NPs) was synthesized and used as control sample. Figure 12 shows the in vitro cytotoxic effect of DS-P-NPs, DS-PPF-NPs, PPF-NPs and free disulfiram on MCF7 and 4T1 cells. The results indicated that both DS-PPF-NPs and DS-P-NPs had higher cytotoxicity compared to free disulfiram. At 500 nM concentration of disulfiram, the cell viability of MCF7 was 68.13 % for free disulfiram compared to 27.6 and 35.6 % for DS-PPF-NPs and DS-P-NPs, respectively (Fig. 12a). For 4T1 cells, the cell viability at 500 nM concentration of disulfiram was 46.15 % for free disulfiram compared to 31.57 and 45.7 % for DS-PPF-NPs and DS-P-NPs, respectively (Fig. 12b).

Also to determine if blank NPs have any cytotoxic effect, MCF7 and 4T1 cells were treated with different concentrations of PPF-NPs (0, 0.6, 1.2, 1.8, 2.4, 3, 4.5, 6, 7.5, 9, 12 μg/ml). The results showed that PPF-NPs did not have any cytotoxic effects neither on MCF7 nor 4T1 cells.

### Acute and chronic toxicity

The in vivo toxicity of DS-PPF-NPs was assessed following of the injections of the drug into mice through acute and chronic toxicity evaluation. The initial given dose was 2000 mg/kg of DS-PPF-NPs which corresponded to 100 mg/kg equivalent of disulfiram. No death or loss of body weight was detected in this group of animals. To evaluate any possible abnormalities in hematological markers of this group, complete blood analysis was performed. For 100 mg/kg group, reduction in SGOT, increase in SGPT, reduction in ALP and direct bilirubin, increase in albumin and total protein, decrease in total leukocyte count and mean corpuscular hemoglobin concentration were observed. Based on these results we decrease the dose of injection to 12.5, 6 and 3 mg/kg equivalent of disulfiram. For 12.5 mg/kg, increase in albumin and total protein and decrease in total leukocyte count and mean corpuscular hemoglobin concentration were detected. No sign of any abnormality in the other groups i.e. 6 and 3 mg/kg equivalent of disulfiram were observed (Table 2). Therefore, the initial dose for chronic toxicity test was chosen as 6, 3, 1.5, and 0.75 mg/kg equivalent of disulfiram from DS-PPF-NPs. To investigate the chronic toxicity of blank NPs (PPF-NPs), the same amount of PPF-NPs (6 mg/kg equivalent of disulfiram) was injected to the control group. The results showed no significant hematological abnormality in any of the animal groups (Table 3).

**Table 2:** Acute toxicity of DS-PPF-NPs in different concentration (control, 3, 6, 12.5 and 100 mg/kg equivalent of disulfiram) on hematological markers in mice

Parameter	Groups				
	Control	DS-PPF-NPs (3 mg/kg)	DS-PPF-NPs (6 mg/kg)	DS-PPF-NPs (12.5 mg/kg)	DS-PPF-NPs (100 mg/kg)
Animal weight (g)	22.3 ± 1.9	22.7 ± 3.6	21.4 ± 2.2	22.9 ± 2.4	23.2 ± 1.8
Urea (mg/dL)	63.3 ± 7.6	75.0 ± 0.0	63.3 ± 10.4	65.0 ± 8.7	64.7 ± 6.5
Cr (mg/dL)	0.5 ± 0.0	0.5 ± 0.0	0.5 ± 0.0	0.5 ± 0.1	0.5 ± 0.1
ALP (U/L)	571.7 ± 92.9	478.3 ± 100.5	470.0 ± 111.7	583.0 ± 91.2	262.0 ± 39.3 ^*^
SGOT (U/L)	396.7 ± 177.9	410.0 ± 91.7	293.3 ± 7.6	396.0 ± 152.7 ^*^	75.0 ± 6.0 ^*^
SGPT (U/L)	41.7 ± 18.9	63.3 ± 15.3	53.3 ± 10.4	65.0 ± 5.0	85.0 ± 6.2 ^*^
D. BIL (mg/dL)	0.5 ± 0.1	0.4 ± 0.1	0.4 ± 0.0	0.4 ± 0.0	0.2 ± 0.0 ^*^
ALB (mg/dL)	2.5 ± 0.0	3.3 ± 0.6	3.0 ± 0.5	5.0 ± 0.0 ^*^	4.7 ± 0.6 ^*^
Total protein (mg/dL)	4.8 ± 0.6	4.5 ± 0.0	4.2 ± 1.5	7.3 ± 0.2 ^*^	6.6 ± 0.3 ^*^
Glucose (mg/dL)	208.3 ± 70.8	271.7 ± 102.1	265.0 ± 98.4	243.0 ± 20.8	183.0 ± 35.1
WBC (1000/mm3)	12.8 ± 3.6	11.2 ± 0.8	12.3 ± 0.9	5.4 ± 0.7 ^*^	3.6 ± 0.2 ^*^
RBC (Millin/mm3)	9.5 ± 0.3	8.7 ± 0.5	8.3 ± 0.5	8.7 ± 0.2	9.5 ± 0.8
HGB (mg/dL)	15.2 ± 0.1	13.9 ± 0.7	13.0 ± 0.9	13.2 ± 0.8	14.4 ± 1.3
HCT (%)	42.5 ± 0.8	44.9 ± 2.3	44.1 ± 2.8	37.2 ± 2.4	39.7 ± 2.8
MCV (FL)	44.6 ± 0.5	52.0 ± 0.5	53.3 ± 0.3	42.8 ± 2.0	42.0 ± 1.6
MCHC (mol/L)	31.7 ± 0.3	30.9 ± 0.5	30.5 ± 0.2	15.2 ± 0.6^*^	15.2 ± 0.5^*^
Plt (1000/mm3)	1114.0 ± 77.8	744.0 ± 127.8	816.3 ± 71.6	1028.7 ± 124.4 ^*^	880.7 ± 55.2 ^*^

The values are mean ± SD

* *p* < 0.05 compared to control

**Table 3:** Chronic toxicity of DS-PPF-NPs in different concentration (control, 0.75, 1.5, 3 and 6 mg/kg equivalent of disulfiram) on hematological markers in mice

Parameter	Groups				
	Control	DS-PPF-NPs (0.75 mg/kg)	DS-PPF-NPs (1.5 mg/kg)	DS-PPF-NPs (3 mg/kg)	DS-PPF-NPs (6 mg/kg)
Animal weight (g)	23.3 ± 2.1	24.3 ± 2.5	22.1 ± 1.8	23.4 ± 1.2	20.2 ± 2.4
Urea (mg/dL)	66.7 ± 2.6	73.3 ± 14.4	75.0 ± 13.5	76.3 ± 10.3	75.0 ± 0.0
Cr (mg/dL)	0.5 ± 0.0	0.5 ± 0.0	0.5 ± 0.0	0.5 ± 0.0	0.5 ± 0.0
ALP (U/L)	505.0 ± 142.0	468.3 ± 93.2	457.5 ± 47.9	452.5 ± 128.5	437.0 ± 70.8
SGOT (U/L)	497.5 ± 101.0	500.0 ± 161.7	490.0 ± 0.0	505.0 ± 86.6	418.3 ± 2.6
SGPT (U/L)	68.8 ± 23.4	70.0 ± 30.9	81.7 ± 11.3	76.7 ± 20.7	60.0 ± 11.5
D. BIL (mg/dL)	0.6 ± 0.1	0.5 ± 0.0	0.5 ± 0.0	0.4 ± 0.1	0.5 ± 0.2
ALB (mg/dL)	2.4 ± 0.2	2.4 ± 0.4	2.3 ± 0.3	2.3 ± 0.3	2.9 ± 0.6
Total protein (mg/dL)	6.3 ± 1.8	5.3 ± 0.3	5.9 ± 0.6	5.5 ± 0.7	6.0 ± 1.2
Glucose (mg/dL)	220.0 ± 57.7	215.0 ± 85.6	197.5 ± 31.8	233.3 ± 6.8	215.0 ± 66.4
WBC (1000/mm3)	6.3 ± 0.1	7.4 ± 1.3	7.2 ± 2.0	7.1 ± 0.8	2.3 ± 7.5
RBC (Millin/mm3)	7.7 ± 0.2	8.2 ± 0.4	8.3 ± 0.2	8.0 ± 0.1	7.6 ± 0.6
HGB (mg/dL)	12.2 ± 0.4	12.1 ± 0.6	12.5 ± 0.3	12.2 ± 0.5	12.4 ± 1.0
HCT (%)	38.2 ± 1.6	38.7 ± 2.2	39.8 ± 1.4	38.5 ± 1.3	40.6 ± 3.3
MCV (FL)	49.3 ± 1.2	47.5 ± 0.4	47.8 ± 0.7	48.1 ± 1.3	52.0 ± 0.0
MCHC (mol/L)	31.8 ± 0.2	31.3 ± 0.8	31.5 ± 0.3	31.6 ± 0.3	27.4 ± 6.0
Plt (1000/mm3)	1028 ± 334.9	943 ± 231	1013 ± 143	1081 ± 327	881 ± 59.3

The values are mean ± SD

* *p* < 0.05 compared to control

### Inhibition of tumor growth in mouse model

**Fig. 13:**
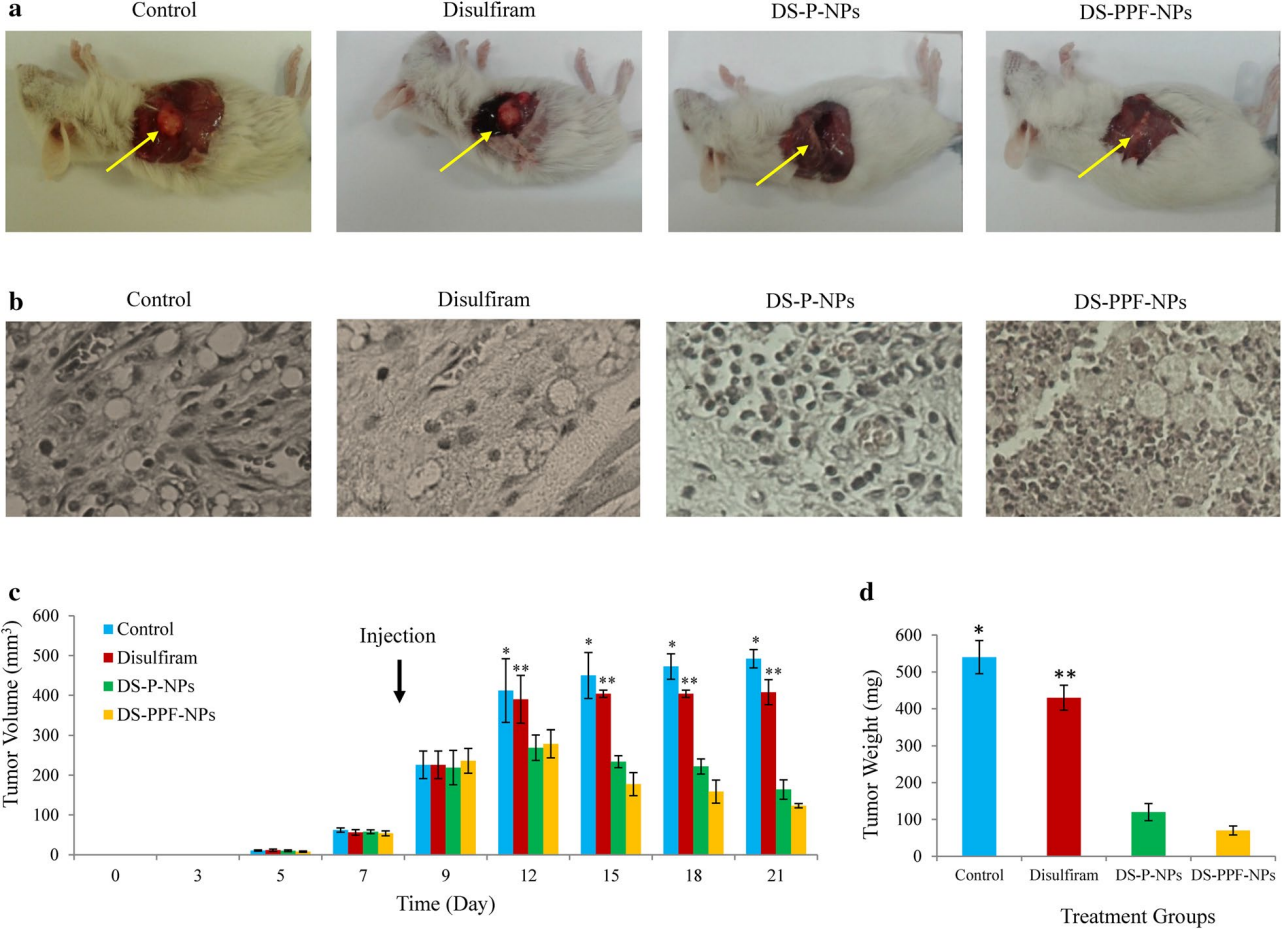
The inhibition of breast cancer tumor growth in BALB/c mice using i.v. injection of blank NPs as control, disulfiram, DS-P-NPs and DS-PPF-NPs (**a**); The histo-pathological image (H&E staining) of tumor from control, disulfiram, DS-P-NPs and DS-PPF-NPs injection groups with 400 times magnification (**b**); the comparison between tumor volume of control, disulfiram, DS-P-NPs and DS-PPF-NPs injection groups (**c**); the comparison between tumor weight of control, disulfiram, DS-P-NPs and DS-PPF-NPs injection groups (**d**); the values are mean ± SD. *Indicates the significant difference between control and NPs (*p* < 0.05); **indicates the significant difference between disulfiram and NPs (*p* < 0.05)

The inhibition of breast cancer tumor growth in BALB/c mice (using 4T1 cells) was performed with 10 mg/kg disulfiram equivalent injection of DS-P-NPs and DS-PPF-NPs into tail vein of animals. The 10 mg/kg of disulfiram equivalent was chosen as the chronic toxicity. The same amount of free disulfiram was dissolved in PBS and injected into tail vein of animals in order to compare the tumor inhibition efficacy of free disulfiram with the DS-P-NPs and DS-PPF-NPs. The results showed that compare to free disulfiram, DS-P-NPs and DS-PPF-NPs significantly inhibited the growth of tumor in mice (Fig. 13a). Compare to the control (blank NPs) and free disulfiram groups, the cancerous cells in IV injection of DS-P-NPs and DS-PPF-NPs show signs of necrotic cells (Fig. 13b).

After 2 weeks of injection, the volume of tumors was 492 ± 23.09 mm^3^ for control group while the tumor size of 123 ± 5.16, 164 ± 24.5 and 408 ± 31.3 mm^3^ was measured for DS-PPF-NPs, DS-P-NPs and free disulfiram injection groups, respectively (Fig. 13c). Besides, no sign of toxicity such as body weight loss was detected in the animals group with DS-P-NPs and DS-PPF-NPs injection.

**Fig. 14:**
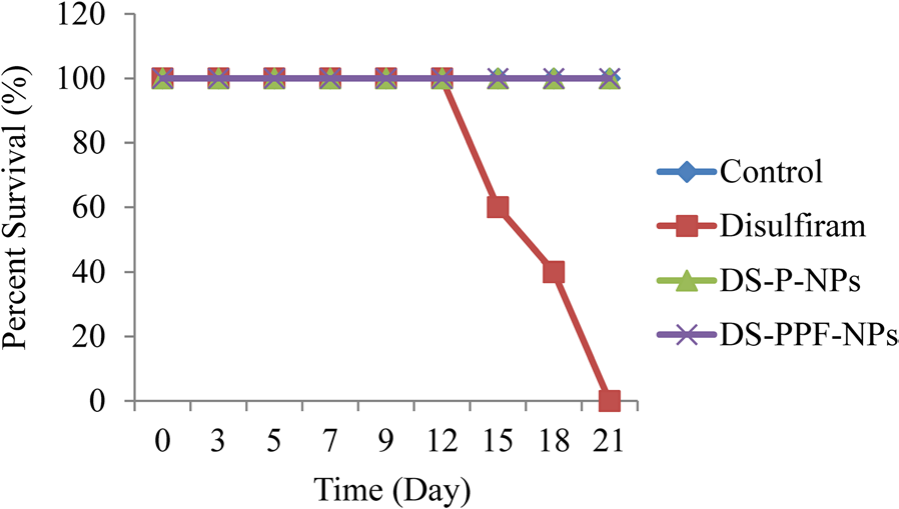
Survival rates of tumor bearing mice treated with PBS (control), disulfiram, DS-P-NPs and DS-PPF-NPs

The tumor weight at the final stage of experiment were measured and the results showed significant reduction in tumor weight in mice with free disulfiram and disulfiram encapsulated NPs injection groups. Compare to free disulfiram, both DS-P-NPs and DS-PPF-NPs injection groups results more reduction in tumor weight (Fig. 13d). The survival rate of mice in disulfiram injection group was reduced compare to control, DS-P-NPs and DS-PPF-NPs (Fig. 14).

## Discussion

In its early discovery, disulfiram considered just as a drug for treatment of alcoholism. Recently with the evidence of its anticancer activity both in vitro and in vivo, this drug recognized as a “repurposing drug” for treatment of hematological malignancy and solid tumors. However, it’s instability in gastric environment and rapid degradation in blood stream hampered its clinical usage for cancer therapy. In this study, disulfiram was encapsulated into folate-receptor-targeted PLGA nanoparticles for its targeted delivery and controlled release into tumor and to inhibit its rapid degradation in blood stream.

Physiochemical properties of NPs such as their size and morphology have important role in their efficiency in drug delivery into tumor site. In solid tumors, several abnormalities could occur during blood vessel formation including deficiency in pericytes and aberrant basement membrane formation. These abnormalities result in leaky vessels with gap size of 220 nm to 1.2 μm between adjacent endothelial cells. The NPs with the size of 220 nm or smaller have access to the tumor that has a higher retention time than normal tissues (EPR effect) [4, 20]. The particle size of <10 nm can help for a faster renal clearance of NPs [4, 20]. The particle size of >220 nm reduce the chance of the NPs to pass through the leaky vasculature system of tumors [4, 20]. Moreover, the larger NPs may eliminate from blood by phagocytic uptake and reticuloendothelial system (RES) [28]. Therefore, the best particle size for delivery purposes into solid tumors should be in the range of 10–220 nm [17, 20]. The nanoprecipitation method for disulfiram encapsulated NPs synthesis resulted in uniform morphology and proper particle size which would facilitate their passage through leaky vessels in tumor tissue using EPR effect. The larger size of DS-PPF-NPs compared to DS-P-NPS might be because of the outside orientation of the PEG-folate moieties on the surface of NPs. The higher drug content and encapsulation efficiency of DS-PPF-NPs could be due to its larger particle size. This is previously reported by other investigations that the PLGA-PEG-folate has larger particle size and higher loading efficiency compared to the PLGA NPs [18]. The rapid initial release of disulfiram from NPs could be due to the adsorbed drug on the surface of NPs and the large surface to volume ratio of them [18]. After the burst release phase, a constant slow drug release profile was observed within the next 96 h of experiment indicating a typical sustained and prolonged drug release which can be correlated to the drug diffusion and matrix erosion mechanisms [18].

The results show that DS-PPF-NPs may have advantage over DS-P-NPs in regards with the uptakes by MCF7 cells. MCF7 is a folate receptor positive cell line which expresses the folate receptor on its surface at a significant level. It seems that folate receptor-mediated endocytosis mechanism may have a role in cellular uptake of DS-PPF-NPs. We used folate to cover the surface of the NPs as the folic acid is small, stable, inexpensive and non-immunogenic molecule. Also, the folate receptor is overexpressed on the vast majority of cancer tissues while its expression is limited in healthy tissues and organs [21]. Folate receptors are highly expressed in epithelial, ovarian, cervical, breast, lung, kidney, colorectal and brain tumors [21]. Approximately, 30 % of breast cancers and 80 % of stage IV metastatic triple negative breast cancer (TNBC) tumors express folate receptor [29].

DS-PPF-NPs induced more apoptosis and therefore has higher cytotoxicity compared to DS-P-NPs. It should be noted that for a similar amount of disulfiram, less DS-PPF-NPs is needed rather than DS-P-NPs for apoptosis induction. It is apparent that DS-PPF-NPs could effectively deliver disulfiram into breast cancer cells (MCF7) and induce apoptosis. Additionally, the higher cytotoxicity of DS-PPF-NPs could be corroborated to the higher affinity of folate conjugated NPs for MCF7 cells [18, 20].The increase of ROS level induced by DS-PPF-NPs in comparison with free disulfiram is an indication that more active disulfiram was transformed into the cells to prompt higher ROS production.

The results of MTT assay show that disulfiram loaded NPs have more cytotoxic effect on breast cancer cell lines (MCF7 and 4T1) compared to free disulfiram. The cytotoxic effect of disulfiram loaded NPs clearly could not be because of the PLGA or PEG as they are both FDA-approved biocompatible polymers. The trace amount of PVA which was used as emulsifier in NPs preparation process and remained in the NPs is not cytotoxic [18]. The higher cytotoxicity effect of disulfiram encapsulated PLGA-NPs could be the result of their internalization into cell by folate receptor mediated endocytosis. It is known that PLGA-NPs could show a higher cellular uptake compared with the free drug itself [18, 20]. The higher cellular internalization of DS-PPF-NPs leads to a higher cellular uptake of the entrapped therapeutic agent [30], enabling them to escape from the effect of P-glycoprotein (P-gp) pumps and thereby showing higher cytotoxic effect compared to free disulfiram as reported previously [30, 31]. Thus, NPs may act as intracellular drug depots that would slowly release the encapsulated therapeutic agent into cellular cytoplasm leading to enhanced therapeutic efficacy of the drug.

The significant decrease in tumor volume would be an indication for efficient tumor growth inhibition caused by drug encapsulated nanoparticles. The observed anti-tumor effect of disulfiram encapsulated PLGA NPs could be attributed to their accumulation into tumor and prevention of the fast drug degradation from the blood stream.

Considering tremendous effects of disulfiram against cancer cells, its robust history of being well-tolerated within human body, and its low price and availability in the market compared to other chemotherapy agents, it is expected that encapsulation of disulfiram into folate-receptor-targeted PLGA-PEG NPs could provide a tool for translation of this drug into clinical cancer therapeutics.
